# CCAAT/Enhancer-Binding Protein **α** Is a Crucial Regulator of Human Fat Mass and Obesity Associated Gene Transcription and Expression

**DOI:** 10.1155/2014/406909

**Published:** 2014-04-29

**Authors:** Wei Ren, Jianjin Guo, Feng Jiang, Jun Lu, Ying Ding, Aimei Li, Xiubin Liang, Weiping Jia

**Affiliations:** ^1^Department of Endocrinology and Metabolism, Shanghai Jiao Tong University Affiliated Sixth People's Hospital, Shanghai Diabetes Institute, Shanghai Key Laboratory of Diabetes Mellitus, Shanghai Clinical Center for Diabetes, Shanghai 200233, China; ^2^Department of Endocrinology, The Second Affiliated Hospital of Shanxi Medical University, Taiyuan, Shanxi 030001, China; ^3^The Center of Metabolic Disease Research, Nanjing Medical University, Nanjing, Jiangsu 210029, China

## Abstract

Several susceptibility loci have been reported associated with obesity and T2DM in GWAS. Fat mass and obesity associated gene (FTO) is the first gene associated with body mass index (BMI) and risk for diabetes in diverse patient populations. FTO is highly expressed in the brain and pancreas, and is involved in regulating dietary intake and energy expenditure. While much is known about the epigenetic mutations contributing to obesity and T2DM, less is certain with the expression regulation of FTO gene. In this study, a highly conserved canonical C/EBP**α** binding site was located around position −45~−54 bp relative to the human FTO gene transcriptional start site. Site-directed mutagenesis of the putative C/EBP**α** binding sites decreased FTO promoter activity. Overexpression and RNAi studies also indicated that C/EBP**α** was required for the expression of FTO. Chromatin immunoprecipitation (ChIP) experiment was carried out and the result shows direct binding of C/EBP**α** to the putative binding regions in the FTO promoter. Collectively, our data suggest that C/EBP**α** may act as a positive regulator binding to FTO promoter and consequently, activates the gene transcription.

## 1. Introduction

Human FTO consists of 505 amino acids, with the mature protein predicted to have a mass of approximately 58.3 kDa. The research of crystal structure confirmed FTO gene encodes a 2-oxoglutarate (2-OG) Fe^2+^-dependent dioxygenase and is expressed widely in human tissues [1]. The functional domain contains several residues that are absolutely conserved among highly diverse species. Previous research on FTO mainly focused on the epigenetics. Several groups have revealed that single nucleotide polymorphisms (SNPs) within the first intron of FTO are strongly associated with adiposity and diabetes by genome-wide association studies (GWAS) [2]. FTO is highly expressed in the hypothalamus and pancreatic islets and widely expressed at a lower level in multiple tissues including adipose tissue, liver, and skeletal muscle. Berulava et al. showed that altered FTO levels affect the transcript of genes related to RNA processing and metabolism [3]. However, the molecular mechanisms responsible for transcriptional regulation of human FTO gene have not previously been completely elucidated.

CCAAT/enhancer-binding proteins (or C/EBPs) are a family of transcription factors, composed of six members called C/EBP*α* to C/EBP*ζ*. They promote the expression of certain genes through interaction with promoter region. Once bound to DNA, C/EBPs can open up chromatin structure or recruit basal transcription factors to regulate gene expression of many housekeeping and tissue-specific genes. C/EBP*α* is required for both adipogenesis and normal adipocyte function [4]. For example, C/EBP*α* is not only necessary but also sufficient to initiate the 3T3-L1 adipocyte differentiation program [5]. In mouse model, obese genes have been reported to be transcriptional activated by C/EBP*α*. Mice lacking C/EBP*α* show abnormal adipose tissue formation [6]. Moreover, ectopic expression of C/EBP*α* in various fibroblast cell lines promotes adipogenesis.

More recently, we have reported that transcription factor Foxa2 negatively regulates human FTO gene promoter, but the positive transcription factor has not been revealed. In the present study, the human FTO gene promoter structure and its transcriptional control elements have been identified. Mutational and functional analysis of the promoter revealed a functional C/EBP*α* binding sequence at positions −45~−54 relative to the transcriptional initiation site in the FTO promoter. siRNA and cotransfection studies indicated that C/EBP*α* upregulates its transcription. C/EBP*α* associates with the binding sites of the FTO gene promoter, as demonstrated in ChIP assays* in vivo*. Thus, our study established a molecular basis for further understanding the mechanisms governing FTO gene expression, by which FTO may act as a regulator to enhance adipogenesis.

## 2. Materials and Methods

### 2.1. Cell Culture and Treatment

The human embryonic kidney 293 (HEK 293) and human cervical carcinoma (HeLa) cells were cell lines obtained from Shanghai Cell Biology Medical Research Institute, Chinese Academy of Sciences. These cells were maintained in DMEM (Invitrogen) containing 10% heat-inactivated fetal bovine serum and 1% antibiotic-antimycotic agents.

### 2.2. Bioinformatics Transcriptional Elements Analyses

To identify transcriptional regulatory sequences and potential transcription factor binding sites on the putative promoter regions, the sequences of human, mouse, and rat were obtained from GenBank and aligned by Clustal X program. We analyzed the 5′-flanking regions at the transcription start site with TFsearch (http://mbs.cbrc.jp/research/db/TFSEARCH.html) and AliBaba 2.1 (http://www.gene-regulation.com).

### 2.3. Transient Transfections and Luciferase Assay

The cloning of the human FTO gene promoter region and the constructing of promoter luciferase report plasmids were performed as described previously [7]. For assaying luciferase expression, HEK 293 and Hela cells were seeded onto 24-well plates, cultured overnight, and cotransfected with pGL3 vector reporter construct and pRL-TK (Promega) as a transfection efficiency control. Cells were lysed and assayed for both firefly and Renilla luciferase using the Dual Luciferase Reporter Assay System Kit (Promega) at 24 h after transfection. Luminescence was determined in a Modulus luminometer (Turner Biosystems) after addition of substrate to allow adequate mixing. Relative firefly luciferase activities (RLU) were calculated by normalizing transfection efficiencies with the Renilla luciferase activity. All the data shown in this study were obtained from at least three independent experiments.

### 2.4. Overexpression of C/EBP*α*

In overexpression experiments, the expression plasmids pcDNA3.1-C/EBP*α* and pcDNA3.1 empty vector were purified and cotransfected by using Lipofectamine 2000 (Invitrogen). Total RNA was isolated 24 hours later and analyzed by RT-PCR. For western blotting experiments, lysates were obtained from cells cultured for 48 hours in 6-well plates.

### 2.5. Small Interfering RNA Transfection

In the RNA interference experiments, HEK293 cells were seeded in 6-well plates 24 h before transfection. Cells grown to 50% confluency were washed once with serum and antibiotic-free medium and transfected with 100 nM C/EBP siRNA using 2 *μ*L Lipofectamine (Invitrogen) in serum-free medium. After 4 h incubation, complete medium without antibiotics was added and cells were incubated for 24 h. siRNAs specifically targeting C/EBP*α* (sense, 5′-GUCGGCCAGGAACUCGUCGTT-3′; and antisense, 5′-CGACGAGUUCCUGGCCGACTT-3′) were custom designed [8]. Scrambled siRNA (sense, 5′-GUAGUCCAUGGACCCGUAGTT-3′; and antisense, 5′-CUACGGGUCCAUGGACUACTT-3′) was used as a negative control.

### 2.6. Site-Directed Mutagenesis

Mutation of the putative C/EBP*α* sites at −45/−54 of human FTO promoter was performed using MutanBEST site-directed mutagenesis kit (Takara) with the pGL3-100 plasmid as the template. The mutagenesis primers designed for the mutations were as follows (the mutated sequences are underlined): mu- C/EBP*α*-Forward: 5′-CCTCCTGAACAATGTAGTTCTC-3′, Reverse: 5′-CTACGGGAGCCTGCCATGTTTC-3′; mu-Sp1-Forward: 5′-GGGGTAATAGACTACGCTCTT-3′, Reverse: 5′-CCGCCGACGACCGGGAACCTAC-3′. The mu-C/EBP*α*-Sp1 plasmid was created using mu-Sp1 as template. In the mutant expression clones, the sequences of the entire region mutated were amplified by PCR and the expected mutations were verified by DNA sequencing.

### 2.7. Real-Time PCR

The indicated plasmids or siRNAs were transfected into cells as described above. Total RNA was isolated according to the standard TRIZOL (Invitrogen) method. First-strand cDNA was synthesized from 1 *μ*g of total RNA using M-MLV reverse transcriptase (Promega). Real-time PCR was performed with ABI system (ABI 7500). The Qiagen 2x SYBR Green master mix was used for PCR reaction. Negative control reactions contained sterilized double-distilled water instead of cDNA and were included in each run to ensure absence of contamination. Thermal denaturation (melt curve analysis) was used to confirm the specificity of desired PCR products. Quantitated mRNA levels of analyzed genes were normalized to GAPDH mRNA to generate a relative expression ratio. Primers utilized were as follows: FTO, forward 5′-ACTTGGCTCCCTTATCTGACC-3′ and reverse 5′-TGTGCAGTGTGAGAAAGGCTT-3′; GAPDH, forward 5′-AGGACTCATGTCCATGCCAT-3′ and reverse 5′-ACCCTGTTGCTGTAGCCAAA-3′.

### 2.8. Western Blot Analysis

After transfection and treatment, cells growing on 6-well cell-culture plates were washed in ice-cold PBS and lysed with 200 *μ*L of lysis buffer containing a protease inhibitor cocktail (Complete; Roche). Protein concentrations were determined by the colorimetric BioRad Protein Assay (BioRad). Protein samples were prepared with 5x SDS sample buffer and loaded at 20 *μ*g of protein per lane for SDS-PAGE. Western blot was performed with FTO (Abgent) and C/EBP*α* (Santa Cruz) antibodies, followed by goat anti-mouse IgG conjugated with HRP. GAPDH was detected as loading control. Chemoluminescence signals from three independent western analyses were quantified using an ECL imager and analyzed using Quantity One software (BioRad).

### 2.9. Chromatin Immunoprecipitation Assays

ChIP assays were performed according to the protocols provided by the manufacturer (Active Motif, Carlsbad, CA). Chromatin DNA was fragmented by sonication to an average length of 0.5 kb. Formaldehyde-fixed DNA/protein complex was immunoprecipitated with 5 *μ*g of normal rabbit IgG, anti-C/EBP*α* antibody (Santa Cruz) and the DNA was purified using gel exclusion columns. The purified ChIP DNA fragment was subjected to semiquantitative PCR analysis (1 cycle of 95°C for 3 min, 35 cycles of 95°C for 20 s, 64°C for 20 s, and 72°C for 1 min). Specific forward (5′-CATTGCTATAGCGCCGACAGCG-3′) and reverse (5′-GAGAATTTCCCAGGTCCGACAG-3′) primers were designed to amplify the FTO promoter region between −133 and +36 bp relative to the transcription start site, which contains C/EBP*α* binding sites. The sample from total chromatin before immunoprecipitation (at 1:10 dilution) was used as positive control. The PCR products were analyzed on a 2% agarose gel and quantified by densitometry using Fluor's fluorimeter and Quantity One software (Bio-rad).

### 2.10. Statistical Analysis

Data are expressed as means ± SEM. Comparisons between means were performed by unpaired two-tailed Student's *t*-test using SPSS software. *P*-value less than 0.05 was considered significant.

## 3. Results

### 3.1. The FTO Promoter Contains a C/EBP*α* Binding Site

**Figure 1:**
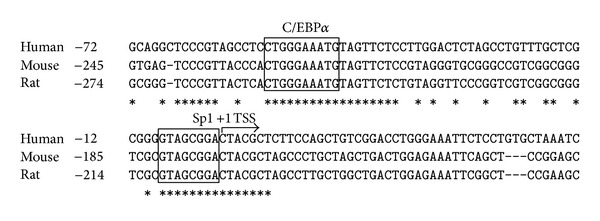
Putative C/EBP*α* binding site is located in FTO gene promoter. Human genomic sequence of FTO gene from −72 to +47 bp is shown. Asterisks indicate positions where the bases are absolutely conserved among the three species. The transcription start site (TSS) is shown as indicated in the figure. The boxed sequences represent the predicted C/EBP*α* (−45/−54) and Sp1 (−1/−8) binding sites based on the results of software.

We have previously reported the preliminary analysis of the functional characteristics of human FTO promoter in HEK293 and Hela cells showed that the core promoter was located within the region of −100 bp relative to the transcription start site [7]. Further analysis of the region showed Foxa2 binding sequence (−26/−14) as a negative regulatory element to the expression of human FTO gene. To identify the positive regulatory transcription factors of FTO, the promoter sequences were subjected to search for transcription factor binding sites. A putative consensus C/EBP*α* binding site (TGGGAAAT at −45/−54 bp) and an Sp1 binding site (GTAGCGGA at −1/−8 bp) were predicted within the FTO core promoter region by TFSEARCH and Alibaba 2.1 program (Figure 1). The sequence TGGGAAAT was almost identical to the C/EBP consensus sequence TTNNGNAAT [9]. Sequence alignment by Clustal X reveals the putative nucleotide sequence for C/EBP*α* and Sp1 is well conserved between the mouse, the rattus, and the human FTO genes obtained from GenBank (Figure 1). The significant conservation across species suggesting C/EBP*α* and/or Sp1 transfactor may regulate FTO transcriptional expression.

### 3.2. C/EBP*α* Element Enhances the Basal Level of FTO Promoter Activity

To investigate further the function of the C/EBP*α* and Sp1 binding sites in the regulation of the human FTO gene, mutants were constructed by site-directed mutagenesis. The reporter gene plasmid pGL3-100 which contains human FTO gene core promoter (−100 to +34) was used as template. The luciferase activity of mutant constructs was measured and compared with that of the wild-type promoter (pGL3-100) using HEK 293 cell line.

**Figure 2:**
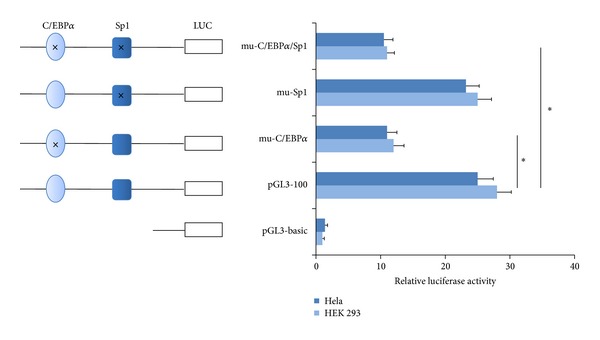
Luciferase reporter assay to determine the regulatory effect of C/EBP*α* on FTO promoter activity. Three mutant variants (mutated at C/EBP site, Sp1 site, and both of them, resp.) were constructed. Relative luciferase activities (RLU) were measured three independent times in Hela and HEK 293 cells. Results are presented as mean RLU ± SE of three independent experiments (*P < 0.05).

The results showed the relative luciferase activities (RLU) of the C/EBP*α* site mutant (pGL3-mu-C/EBP*α*) decreased by 57.1% (Hela cell) or 55.6% (HEK 293 cell) compared with that of pGL3-100. In contrast, the change of luciferase activity is virtually absent in the reporter construct pGL3-mu-Sp1 containing the mutant Sp1 binding site. Furthermore, the two separate site-specific mutagenesis pGL3-mu-C/EBP*α*/Sp1 behaved similarly to the pGL3-mu-C/EBP*α*; luciferase activity was suppressed to 39.3% (Hela cell) or 41.7% (HEK 293 cell) (Figure 2).

### 3.3. C/EBP*α* is Involved in the Expression of FTO

**Figure 3:**
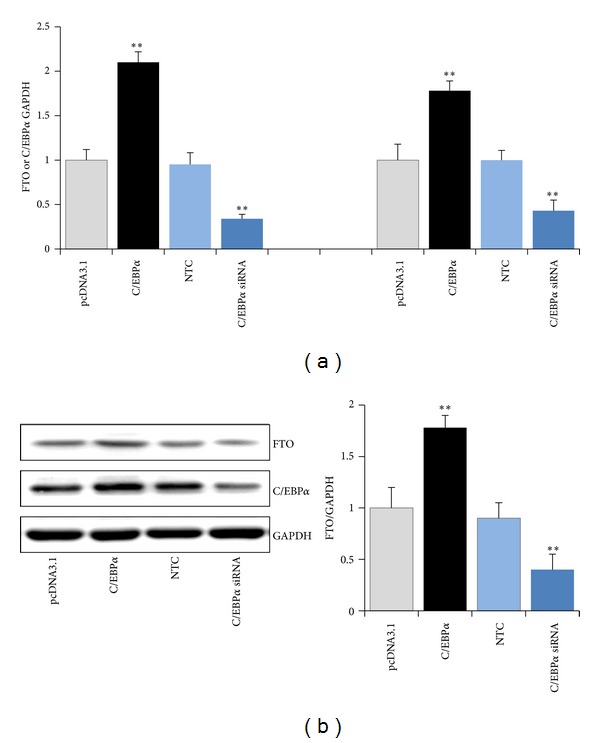
Effect of C/EBP*α* on FTO expression. HEK 293 cells were transfected with pcDNA3.1-C/EBP*α* or C/EBP*α* siRNA. FTO and C/EBP*α* mRNA and protein levels were normalized by their respective GAPDH values. Bars are the mean of three independent experiments ± S.D. (**P < 0.01).

The C/EBP*α* transfactor binding sites have been identified to play a role in regulating the activity of human FTO promoter. In the following experiments, we further examined the ability of C/EBP*α* to modulate the expression of FTO. C/EBP*α* expression vector pcDNA3.1-C/EBP*α* or C/EBP*α* siRNA was transfected into HEK 293 cells. FTO mRNA and protein levels were detected by RT-PCR or Western blotting, respectively. As illustrated in Figure 3, more robust overexpression of C/EBP*α* increased the FTO transcript level 2.1-fold (Figure 3(a)) and FTO protein level by 78.2% above control (Figure 3(b)). Conversely, RNAi-mediated reduction of C/EBP*α* significantly inhibited the FTO expression at both the mRNA level (66.3%) and protein level (56.2%).

### 3.4. C/EBP*α* Binds to the FTO Promoter* In Vivo*

**Figure 4:**
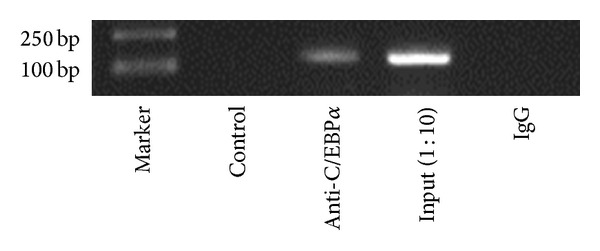
Identification of the sequence motif responsible for activation of the FTO promoter by C/EBP*α*. ChIP assay in HEK 293 cells showing that C/EBP*α* can bind to the human FTO promoter site. Amounts of coprecipitated DNA (Anti-C/EBP*α*) and the corresponding amounts in the input chromatin samples (input 1 : 10) were measured by PCR.

ChIP analysis was performed to test if C/EBP*α* binds to the FTO promoter. Nuclear lysates prepared from HEK 293 were subjected to sonication to shear DNA to lengths between 200 and 1000 bp on ice followed by phenol/chloroform extraction to recover protein/DNA complexes. C/EBP*α* antibody was used to pull down the complexes as instructed. The resultant precipitates were then used as templates for PCR amplification of FTO promoter sequence containing the C/EBP*α* binding motif. As shown in Figure 4, precipitates resulting from C/EBP*α* antibody yielded a corresponding band as that of amplification with input (1 : 10), an aliquot of chromatin that was not incubated with an antibody, while no band was showed in the negative control (IgG). These results clearly demonstrate an* in vivo* recruitment of C/EBP*α* binding element on the human FTO promoter. Taken together, our results suggest that C/EBP*α* specifically binds to the predicted motifs to regulate FTO transcription and expression directly.

## 4. Discussion

The association of the genetic variants of FTO gene with obesity and diabetes was recently identified by several independent GWA studies. Subsequent studies have revealed the influence of FTO variants on measures of appetite, food intake, or energy expenditure. FTO is highly expressed in the brain (hypothalamic) and pancreas. The putative influence of FTO in hypothalamic-pituitary-thyroid axis has been determined [10, 11]. A number of studies have been observed the expression of Fto in hypothalamic correlates with changes in the nutritional status of animals, and furthermore, the abundant hypothalamic expression of FTO also supports a potential role in the control of satiety or appetite [12]. Although these studies reveal the connection between FTO expression and energy metabolism, the molecular mechanism for regulating FTO gene expression remains unclear.

The human FTO gene transcription has been studied previously by our group. We had reported that the region (−100/+34) seems to be crucial because deletion of this fragment would not remain as the basal promoter activity. Transfactor Foxa2 was defined to regulate the transcription and expression of FTO gene negatively. There are no data about transcription factors positively involved in FTO gene expression. Here, we improved the regulatory mechanism of human FTO gene expression. A conserved C/EBP*α* binding sequence was identified in the core promoter region of the FTO gene, suggesting that the binding sites may play important roles in regulating the expression of FTO gene. Promoter-reporter gene constructs that contain the proximal C/EBP binding site showed that C/EBP*α* strongly activates reporter gene expression. Moreover, mutation of the C/EBP*α* binding site within the promoter completely blocked transactivation by C/EBP*α*. We then performed gene transfection-mediated overexpression and RNA interference- (RNAi-) mediated gene silencing of C/EBP*α* on HEK 293 cells and the results suggested that C/EBP*α* promoted the expression of FTO. Consistent with this, ChIP experiments showed that C/EBP*α* binds to the predicted binding element in the FTO promoter. Taken together, these findings provide compelling evidence that C/EBP*α* to be a positive transcriptional factor contributes to the transcription and expression regulation of human FTO gene.

The C/EBP transcription factors consist of six members (*α*, *β*, *δ*, *γ*, *ε*, and *ζ*), containing conserved DNA binding domain at C-terminus and an activation domain at their N-terminus. Data presented that different C/EBP number occurs at different point-in-time in adipogenesis, indicative of their own distinct roles during the progress [13]. C/EBP*α* is expressed in liver, adipose tissue, and muscle and is required for both adipogenesis and normal adipocyte function. Ectopic expression of C/EBP*α* in various fibroblast cell lines promotes adipogenesis. Moreover, it was reported that C/EBP*α* promotes adipogenesis by inducing the expression of peroxisome proliferator activated receptor *γ* (PPAR*γ*). It was reported that PPAR*γ* was able to promote adipogenesis even in the absence of C/EBP*α*, whereas C/EBP*α* could not promote adipogenesis in the absence of PPAR*γ* [14, 15]. Previous research has also shown that in the early phase of 3T3-L1 cell differentiation, FTO expression was transiently increased; however, partial reduction of FTO did not impact PPAR*γ* expression and adipocyte differentiation [16]. Thus, one can speculate that C/EBP*α* mediates different signaling pathways involved in adipogenesis. While some researchers have found that FTO expression was not modulated during differentiation of preadipocytes into mature adipocytes, some have even demonstrated that FTO expression is decreased during differentiation of primary preadipocytes isolated from human subcutaneous adipose tissue or preadipocytes derived from Simpson-Golabi-Behmel syndrome (SGBS) [17, 18]. These results somewhat contrast with each other suggesting that FTO expression and functions are not entirely clear in the different cell types and tissues.

FTO has also shown a positive association with the expression of adiponectin, an anti-inflammatory adipokine in adipose tissues. NF-*κ*B and C/EBP family can be activated by inflammatory cytokines such as interleukin-1 (IL-1) and IL-6, respectively, whereas IL-6 suppresses the transcription of adiponectin [19–21]. Much more rigorous research needs to be conducted to understand the regulation mechanism as in the inflammatory reaction of adipose tissue.

In summary, we have confirmed so far that C/EBP*α* and Foxa2, respectively, positively and negatively regulated the expression of human FTO gene. Given the complex interacting network of transcription factors involved in time-space characteristics of gene expression, it can often be difficult to determine which factor plays a crucial role in the transcriptional regulation process. Data derived from further experiments would unravel the details of FTO functions and regulatory networks.
